# Ganglioside Antibodies as Formidable Neurologic Mimickers: A Case Report of Dual Anti-GQ1b and Asialo-GM1 Antibody Syndrome

**DOI:** 10.7759/cureus.114173

**Published:** 2026-08-08

**Authors:** Emily Ahner, Khalid Haikal, Sannah Vasaya, Paul Janda, Aroucha Vickers

**Affiliations:** 1 Medicine, University of Nevada Las Vegas School of Medicine, Las Vegas, USA; 2 Neurology, Valley Hospital Medical Center, Las Vegas, USA; 3 Neurology, Las Vegas Neurology Center, Las Vegas, USA

**Keywords:** albuminocytologic dissociation, asialo-gm1 antibodies, facial nerve palsy, ganglioside antibody neuropathy, intravenous immunoglobulin (ivig)

## Abstract

The neurologic manifestations of anti-GQ1b antibody syndrome continue to expand and include a spectrum of immune-mediated neuropathies such as Miller Fisher syndrome, Bickerstaff brainstem encephalitis, and optic neuropathy. Asialo-GM1 antibodies are associated with motor or sensorimotor neuropathies, particularly multifocal motor neuropathy. Reports describing concurrent seropositivity for anti-GQ1b and asialo-GM1 antibodies presenting with unilateral facial weakness and limb symptoms remain scarce.

A 55-year-old woman with chronic back pain initially presented to an outside facility with new-onset left lower motor neuron facial palsy accompanied by paresthesia and extremity weakness. Neuro-axis MRI demonstrated moderate-to-severe multilevel spinal and neuroforaminal stenosis. She was discharged with prednisone and acyclovir for presumed idiopathic Bell’s palsy. Shortly thereafter, she presented to our institution with worsening symptoms. Examination revealed diminished strength, hypoesthesia, and decreased reflexes with a persistent left lower motor neuron cranial nerve VII palsy. The remainder of the cranial nerve examination was normal, and there was no ophthalmoplegia or ataxia. Lumbar puncture demonstrated albuminocytologic dissociation. A ganglioside antibody panel was positive for anti-GQ1b and asialo-GM1 antibodies.

This case highlights an unusual presentation of concurrent anti-GQ1b and asialo-GM1 antibody positivity manifesting as unilateral facial palsy and limb weakness initially attributed to Bell’s Palsy. Recognition of ganglioside antibody syndromes as potential mimickers of Bell’s palsy is essential to ensure prompt diagnosis and treatment, thereby reducing the risk of neurological morbidity.

## Introduction

Gangliosides are sialic acid-containing glycosphingolipids that are found in the nervous system [1]. They function to repair neuronal cells, aid in synaptic transmission, and are implicated in a variety of neurological pathologies. Antibodies directed against gangliosides such as GM1, GD1a, and GQ1b disrupt neuronal conduction through complement-mediated injury of axonal and nodal membranes. Their pathogenic activity underlies several overlapping clinical entities, including syndromes associated with anti-GQ1b and asialo-GM1 antibodies [2].

GQ1b is a ganglioside present throughout the nervous system and found in cranial nerves III, IV, and VI; muscle spindles; and dorsal root ganglia [3]. Anti-GQ1b antibody syndrome refers to a spectrum of immune-mediated neuropathies sharing a common autoantibody against the ganglioside GQ1b. A history of prodromal infection is common among patients with anti-GQ1b antibody syndrome [2]. Clinical manifestations of anti-GQ1b syndrome include ophthalmoplegia, hypersomnolence, ataxia, bulbar palsy, and weakness [2]. The neurologic manifestations of anti-GQ1b antibody syndrome continue to expand and include a variety of immune-mediated neuropathies such as Miller Fisher syndrome, Bickerstaff’s brainstem encephalitis, and optic neuropathy [3,4]. The classic phenotype of Miller Fisher syndrome involves a triad of ophthalmoplegia, ataxia, and areflexia. Bickerstaff’s brainstem encephalitis is differentiated from Miller Fisher syndrome and other anti-GQ1b antibody syndromes by the additional symptoms of altered sensorium. Altered sensorium can present as drowsiness, stupor, or coma [5]. These pathologies are recognized as points along a clinical spectrum, involving many overlapping signs and symptoms between cases [2]. Pathology is thought to arise through a process of molecular mimicry between microbial epitopes, for example, *Campylobacter jejuni* or *Haemophilus influenzae*, and the GQ1b ganglioside on neuronal membranes. This resulting cross-reactivity triggers complement-mediated injury of the motor and sensory fibers [2]. The overall prognosis is favorable, with most patients achieving near-complete recovery with appropriate therapy within weeks to months [2]. However, the clinical presentation found in antiganglioside antibody syndromes of cranial neuropathies, peripheral weakness, and sensory syndromes can mimic common diagnoses such as idiopathic Bell’s palsy, degenerative spinal disease, and other Guillain-Barré syndrome variants, leading to a potential delay in the recognition of underlying immune-mediated processes. 

Asialo-GM1 antibodies represent another immunologic marker within the antiganglioside family. Asialo-GM1 is a neutral glycolipid structurally related to GM1. Asialo-GM1 antibodies are linked to motor or sensorimotor neuropathies, including Guillain-Barré, multifocal motor neuropathy, and motor neuron disease [6]. Reports of concurrent seropositivity in patients with unilateral facial weakness, limb weakness, and paresthesia remain scarce [7, 8]. Clinically, motor neuropathies like multifocal motor neuropathy are characterized by asymmetric distal limb weakness without sensory loss and multifocal motor conduction block outside the typically expected sites of nerve compression [9]. Although anti-GQ1b and asialo-GM1 antibodies have distinct clinical phenotypes on their own, concurrent seropositivity may obscure these known presentations and create a diagnostically challenging overlap. Given the previously mentioned clinical overlap, concurrent anti-GQ1b and anti-asialo-GM1 seropositivity in a patient with unilateral facial weakness, limb weakness, and paresthesias suggests an unusual overlap of antiganglioside-mediated mechanisms, expanding upon the recognized spectrum of immune-mediated neuropathies and underscoring the value of broader antibody panels in atypical cases.

This article was previously presented as a meeting abstract at the 2025 American Academy of Neurology on April 6, 2025.

## Case presentation

**Figure 1:**
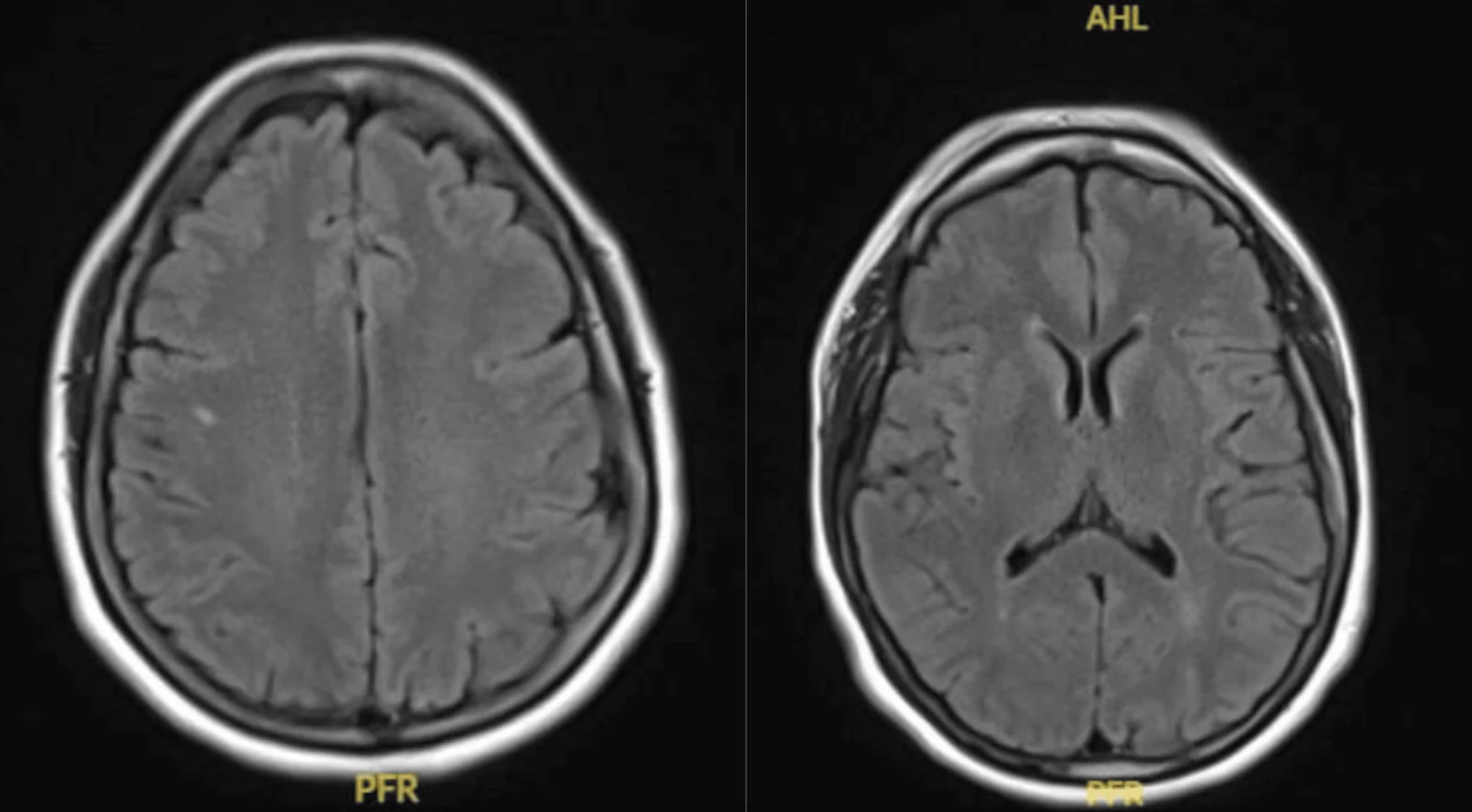
Axial fluid attenuated inversion recovery (FLAIR) MRI of the brain demonstrating mild scattered nonspecific white matter hyperintensities.

**Figure 2:**
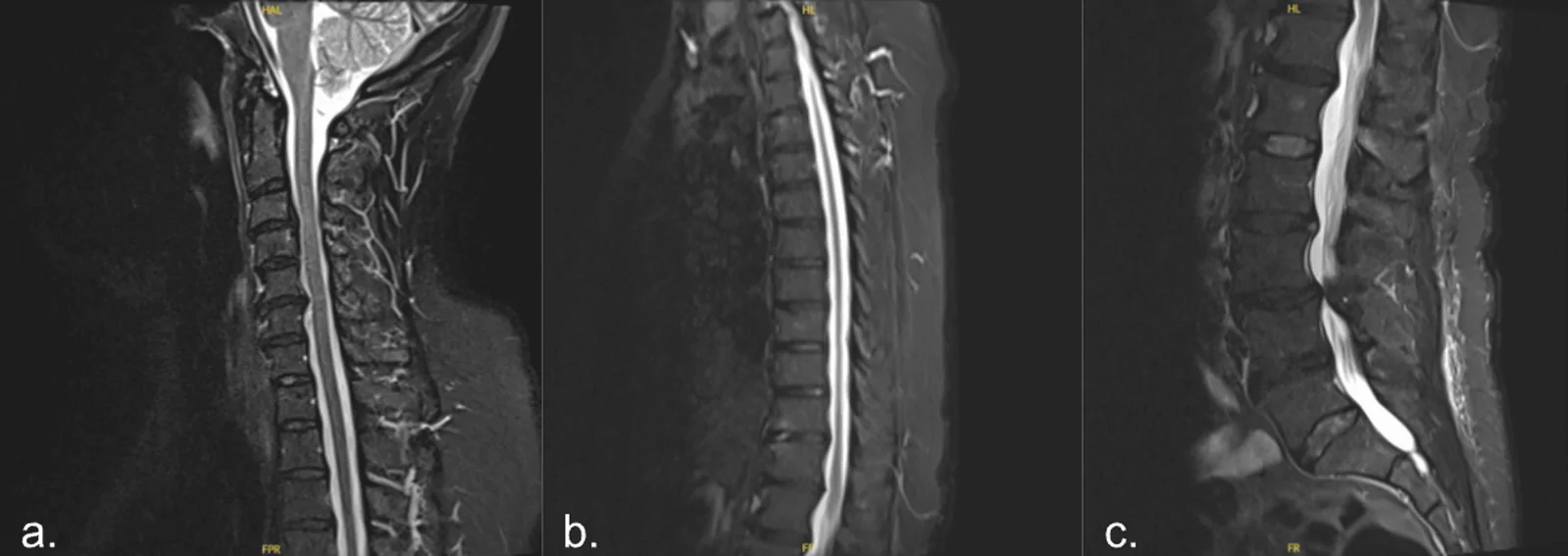
Sagittal short tau inversion recovery (STIR) MRI of the cervical (a), thoracic (b), and lumbar (c) spine demonstrating moderate-to-severe multilevel degenerative changes throughout the spine, including severe L3–L4 spinal canal stenosis and grade 1 L4–L5 spondylolisthesis.

A 55-year-old woman presented to an outside facility with new-onset left facial droop, involving both the upper and lower face, progressive paresthesia, and weakness in her extremities as well as neck and back pain. Her past medical history was significant for prediabetes and chronic spinal stenosis previously managed with physical therapy and steroid injections. Neurologic examination demonstrated a left lower motor neuron facial nerve (cranial nerve VII) palsy, generalized weakness in all extremities with Medical Research Council (MRC) grade 4/5 strength throughout, and bilaterally diminished patellar reflexes (1+). The combination of a lower motor neuron cranial nerve VII palsy, diffuse symmetric weakness involving all four extremities, sensory symptoms, and hyporeflexia suggested a peripheral neuropathic process. This distribution could not be explained by focal lumbar stenosis or multilevel degenerative spinal disease in the absence of spinal cord compression. Brain MRI demonstrated mild scattered white matter lesions as seen in Figure 1. Spinal MRI showed moderate to severe multilevel degenerative changes, noted on cervical, thoracic, and lumbar spine MRI, including severe L3-L4 stenosis with grade 1 spondylolisthesis at L4-L5 as seen in Figure 2. She was started on a seven-day course of prednisone and intravenous acyclovir due to concern for Bell’s palsy and a trial of pregabalin for symptomatic relief. Neurosurgery was consulted and recommended no immediate surgical intervention due to a lack of acute cord compression. She was advised to follow up with her outpatient surgeon for possible elective surgical intervention.

**Figure 3:**
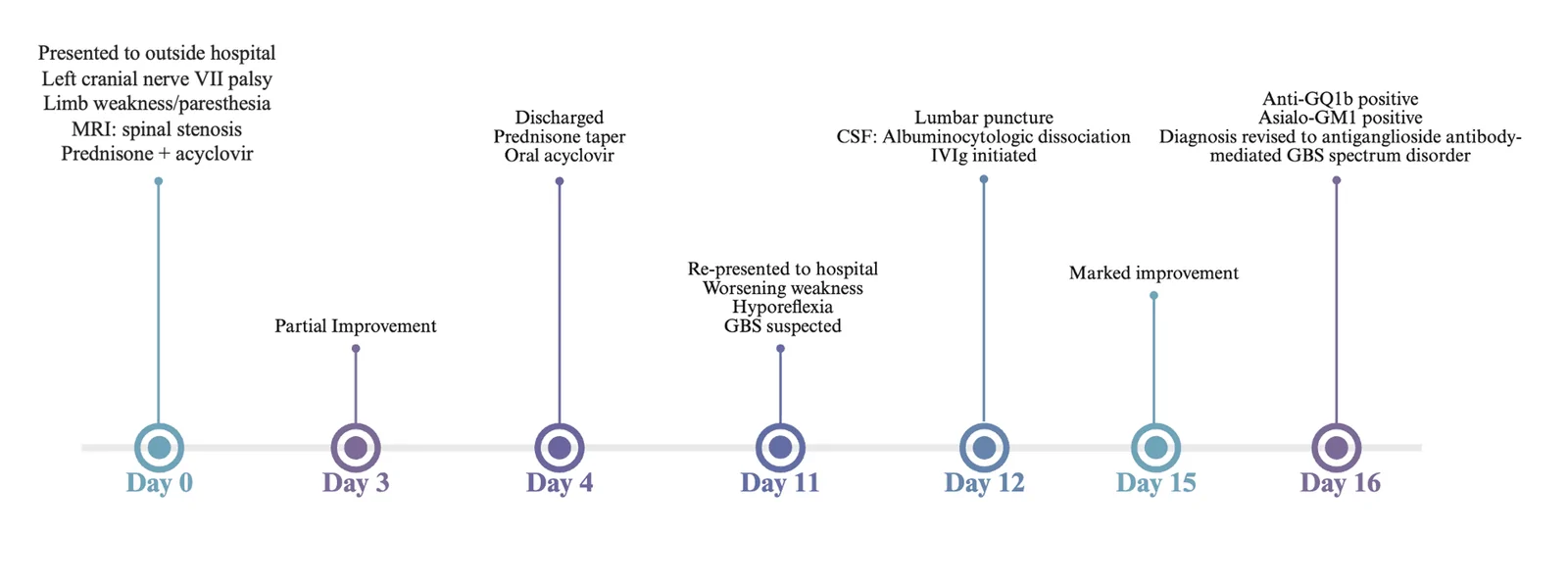
Clinical timeline Clinical timeline demonstrating the patient’s progression from their initial presentation of presumed Bell’s palsy to the eventual antiganglioside antibody-mediated Guillain-Barré syndrome (GBS) spectrum disorder. Despite initial improvement under corticosteroid therapy, progressive symptoms led to cerebrospinal fluid (CSF) analysis and discovery of albuminocytologic dissociation and positive ganglioside antibody testing, leading to initiation of intravenous immunoglobulin (IVIg) treatment and subsequent clinical improvement.

As shown in Figure 3, on day 3, the patient reported clinical improvement in her facial droop and symptomatic relief of paresthesias. She remained on intravenous acyclovir and steroids, then later was transitioned to an oral prednisone taper. On day 4, she was discharged home with continued acyclovir and prednisone taper for seven days, as well as pregabalin and a diagnosis of left-sided idiopathic Bell’s palsy. At discharge, she demonstrated partial resolution of facial weakness. She was instructed to follow up with neurology and spine surgery.

On day 11, she returned to the hospital and presented to our facility due to deteriorating symptoms. She reported persistent and worsening generalized weakness and paresthesias. She reported exposure to “black mold” approximately one month earlier, followed by fever and cough. Approximately one week following this episode, she developed diffuse numbness. Examination revealed 4/5 strength throughout, improving to 4+/5 with improved effort. Cranial nerve II-XII grossly intact except for a left lower motor neuron cranial nerve VII palsy (House-Brackmann II) [10]. Bilateral patellar reflexes were measured at 1/4; otherwise reflexes were 2/4 throughout. There was no ophthalmoplegia or ataxia, and subjectively reduced sensation to light touch was noted throughout. Initial workup included metabolic labs showing elevated glucose, blood urea nitrogen/creatinine ratio, alkaline phosphatase, and leukocytosis. In an effort to rule out an acute structural etiology in the setting of known diffuse degenerative disc disease, neuroimaging was obtained, again demonstrating severe multilevel spinal stenosis without acute cord compression.

On day 12, following the end of the patient’s prednisone and acyclovir course, the patient was started on a five-day course of intravenous immunoglobulin (IVIg), given her progressive generalized weakness, sensory symptoms, hyporeflexia, and concern for a GBS-spectrum disorder. Although IVIg was initially planned on day 11 based on the clinical suspicion for GBS, treatment began on day 12 following fluoroscopy-guided lumbar puncture at the patient’s request. As seen in Table 1, the results of the CSF analysis uncovered an albuminocytologic dissociation with elevated protein concentration (123.6 mg/dL) and no pleocytosis. The meningitis/encephalitis panel came back negative. These findings supported a diagnosis within the Guillain-Barré syndrome spectrum. At this point, a five-day course of IVIg was initiated.

**Table 1:** Cerebrospinal fluid results RBC: red blood cells; WBC: white blood cells

Parameters	Patient Value	Reference Range
Opening pressure	230 mmH_2_O	50-200 mmH_2_O
Color	Clear and colorless	Clear
RBC Count	8 /mm^3^	0 or <1 /mm^3^
WBC Count	0 /mm^3^	0-5 /mm^3^
Glucose	80 mg/dL	50-80 mg/dL
Protein	123.6 mg/dL	15-40 mg/dL

By day 15 (day 4 of IVIg), the patient reported marked improvement regarding her facial weakness and paresthesias, still with some persistent decreased sensation with a House-Brackmann score of II recorded [10]. Further testing, including ANA and heavy metal screens, came back negative.

Following the completion of IVIg, on day 16, the patient reported continued symptomatic improvement. Serum testing was significant for positive anti-GQ1b and asialo-GM1 antibodies, consistent with a diagnosis within the spectrum of GBS with cranial nerve involvement. Ganglioside antibody testing was performed using an immunoassay, which measured both IgG and IgM antibodies against multiple gangliosides. Combined anti-asialo-GM1 IgG/IgM antibodies were positive at 98 IV (reference range, 0-50 IV), corresponding to a positive result (51-100 IV). Combined anti-GQ1b IgG/IgM antibodies were also positive at 94 IV, while the anti-GQ1b IgG antibody was 163 IV, corresponding to a strong positive result (≥101 IV); anti-GQ1b IgM was negative (5 IV). All other tested ganglioside antibodies, including GM1 (combined, IgG, and IgM), GM2, GD1a, and GD1b (combined, IgG, and IgM), were negative. These findings confirmed concurrent anti-GQ1b and asialo-GM1 antibody seropositivity. At the time of reassessment, she demonstrated partial resolution of facial palsy, improved strength, and stable vital signs. She was given instructions to follow up with neurology as an outpatient for electromyography and nerve conduction studies.

## Discussion

The patient's simultaneous lower motor neuron facial palsy, diffuse symmetric limb weakness, sensory symptoms, and hyporeflexia localize to a peripheral neuropathic process rather than focal structural spinal disease. The initial presentation with unilateral lower motor neuron facial palsy, limb weakness, paresthesias, and imaging evidence of multilevel spinal stenosis initially gave the impression of more common diagnoses such as idiopathic Bell’s palsy and degenerative spinal pathology. However, when targeted treatment for these etiologies failed to resolve her symptoms, further testing ultimately supported an immune-mediated antiganglioside pathology. 

Although neuroimaging did demonstrate moderate to severe multilevel spinal stenosis, her symptoms were deemed unlikely to have been caused by a spinal etiology. This assessment was based on a couple of key factors. First, despite the extensive degenerative changes, it was determined that there was no acute spinal cord compression or focal nerve root compression correlating with the patient’s diffuse and symmetric weakness. The combination of generalized weakness involving all extremities, diminished reflexes, and a left lower motor neuron cranial nerve VII palsy could not be localized to a single spinal level or nerve root distribution. Taken together, the discordance between the patient's clinical presentation and spinal imaging findings prompted further evaluation for an alternative neurologic etiology. Although degenerative spinal disease is highly prevalent in older adults, in this case, spinal stenosis represented an important incidental finding rather than the cause of her symptoms. The persistence of cranial neuropathy together with diffuse limb weakness, sensory symptoms, and hyporeflexia further supported a peripheral polyradiculoneuropathic process rather than a focal compressive spinal lesion. 

A limitation of this case is that nerve conduction studies and electromyography were not performed during the patient’s hospital stay, precluding electrodiagnostic characterization of the underlying neuropathy and definitive differentiation between AIDP and other Guillain-Barré syndrome variants; therefore, the diagnosis was based on the clinical presentation, CSF findings, ganglioside antibody profile, and favorable response to IVIg.

The nature of the patient’s symptoms raised reasonable suspicions for Guillain-Barré syndrome, given the progressive weakness, paresthesias, and hyporeflexia. The presence of albuminocytologic dissociation on cerebrospinal fluid analysis also provided strong evidence for a diagnosis within the Guillain-Barré syndrome spectrum. While classic Guillain-Barré syndrome often presents with symmetric limb weakness and areflexia, cranial nerve involvement, particularly facial nerve palsy, can also occur. 

Notably, the patient lacked several classic clinical features found in anti-GQ1b-associated syndromes, including ophthalmoplegia and ataxia, which highlights the heterogeneity of antiganglioside neuropathies and contributes to the difficulty in diagnosis [3,4]. This unique presentation underscores the importance of considering a broad range of immune-mediated neuropathies despite an absent classic presentation, particularly when the symptoms are progressive and multifocal. 

A further complexity added to this case was the administration of high-dose corticosteroids due to a presumed Bell’s palsy diagnosis. While corticosteroids are standard first-line therapy for idiopathic facial nerve palsy, they have been associated with worse outcomes and delayed recovery [11,12]. Although it remains unclear whether steroid exposure contributed to the patient’s symptom progression, her clinical deterioration following initial improvement reflects the progression of underlying immune-mediated neuropathy. This highlights an important precaution that should be exercised when differentiating between Bell’s palsy and other etiologies. 

The concurrent detection of anti-GQ1b and asialo-GM1 antibodies in this patient is both rare and notable. Anti-GQ1b antibodies are classically associated with Miller Fisher syndrome, Bickerstaff’s brainstem encephalitis, and related overlap syndromes, whereas asialo-GM1 antibodies are more commonly linked to motor or sensorimotor neuropathies such as multifocal motor neuropathy and variants of GBS [3,13]. Concurrent seropositivity as seen in this patient provides a different clinical picture than previously recognized for the pathogenesis of ganglioside antibody seropositivity. The case therefore expands the recognized clinical spectrum of antiganglioside antibody-mediated neuropathies and supports the use of comprehensive ganglioside antibody panels in patients with unexplained or mixed neurologic features.

The marked improvement noted after the initiation of the IVIg further supports an immune-mediated etiology. IVIg remains first-line therapy for GBS and related antiganglioside syndromes. Its therapeutic effects are thought to result from multiple immunomodulatory effects, including neutralization of pathogenic antibodies, inhibition of complement activation, and modulation of Fc receptor signaling [14,15]. This case underscores the value of integrating careful neuroanatomic localization with targeted serologic testing to identify atypical antiganglioside antibody syndromes and avoid diagnostic anchoring.

## Conclusions

This case report aims to highlight the diagnostic challenges posed by concurrent GQ1b and asialo-GM1 antibody seropositivity, which mimicked Bell’s palsy in a patient with unilateral facial palsy and limb weakness. Through the characterization of this patient’s clinical presentation, we emphasize the importance of considering ganglioside antibody syndromes in patients with atypical or progressive neurological symptoms. Early recognition and targeted immunotherapy can significantly improve outcomes and prevent unnecessary delays in treatment. As the spectrum of GQ1b antibody syndrome broadens, we report a unique case of GQ1b positivity with asialo-GM1 antibodies, presenting as unilateral facial palsy and limb weakness initially misattributed to other etiologies. This case highlights the importance of clinicians recognizing the potential link between ganglioside antibodies and presentations that mimic Bell’s palsy. Maintaining a high index of suspicion for such formidable mimickers can help mitigate morbidity.
